# Validation of “*creactability*” scale in football: a Rasch modeling approach

**DOI:** 10.3389/fspor.2025.1521073

**Published:** 2025-08-26

**Authors:** Jhinyi Shin, Jongwon Kim, Miyoung Lee, Won Jae Lee

**Affiliations:** ^1^ Department of Physical Education, Chonnam National University, Gwangju, Republic of Korea; ^2^ Department of Data Science, BEPRO Inc., Seoul, Republic of Korea; ^3^ Department of Sport, Health, and Rehabilitation, Kookmin University, Seoul, Republic of Korea; ^4^ Department of Sport Industry and Leisure, Kookmin University, Seoul, Republic of Korea

**Keywords:** item response theory, athlete, quickness, creativity, adaptability

## Abstract

**Purpose:**

The aim of this study was to investigate the construct-related validity evidence of the “*creactability*” scale, developed for athletes in the sports field, using the Rasch model. The specific aims were (1) each scale measures a unidimensional construct, (2) the scale items investigate the Differential Item Functioning (DIF) base on team group (i.e., the rankings of higher and lower teams), (3) the range of “*creactability*” item difficulty and personal ability (respondents “*creactability*” levels) are wide enough to assess the “*creactability*” changes, and (4) “*creactability*” differs across subscales (7 points).

**Methods:**

For Rasch analysis, data from a total of 241 football players were used from the K-League (Korean Professional Football League), excluding goalkeepers due to their different abilities and roles from other field players. The coaches from 7 teams evaluated their players (34.4 players on average per team). The “*creactability*” scale included a total of 9 items, consisting of 3 items each for subfactors (i.e., quickness, creativity, and adaptability) with a 7-point Likert scale. The Rasch model was applied using WINSTEPS Version 5.7.4 and FACETS Version 4.1.8.

**Results:**

The results (1) Confirmed the unidimensionality of all 9 items, as their infit and outfit values fell within the range of 0.7–1.3. (2) None of the items in the position scales showed statistically significant DIF (*p* > 0.05). (3) The Person Separation Index (PSI) criterion value for the “*creactability*” scale of football players is 4, which was within a range of 0–7 point. (4) All subscales demonstrated good-fit in both infit and outfit, ranging between 0.69 and 1.28, respectively. The response rate for scales 4–5–6 point was 68%, and the outfit for respondents across all scales were satisfactory at 1.30 or lower.

**Conclusion:**

Physical activity (PA) should be maintained or increased, particularly in the context of social distancing measures during the pandemic. To ensure that PA can be sustained, a program should be developed that considers the individual's geographical location, economic status, lifestyle, and environment.

## 1 Introduction

The ability to make creative decisions is crucial in sports games (1), as well as in other fields such as music, art, and science (2–4). In football, quick decision-making and creativity are particularly important due to the dynamic nature of the sport, where 22 players consistently interact. The creativity allows players to deal more effectively with unique situations while making it hard for opposition to predict what they will do next (1). Creativity is defined as the player's ability to generate various solutions that are not only original but also appropriate and useful (4, 5). From a tactical standpoint, creativity involves generating diverse solutions to problems in specific situations, characterized by their surprise and rarity (6). Therefore, creativity is associated with a player's ability to make decisions in a specific context, making opponents less likely to anticipate their moves (7).

In the meantime, creativity has not been well utilized as an evaluation factor when assessing the performance of players (teams) in football. Creativity is generally deemed to intangible quality, which is impossible to be assessed through statistics (8). While the conventional concept of creativity is appropriate for explaining works of art, such as paintings, sculptures, poems, and songs, it falls short when applied to improvisational performing arts like jazz, freestyle rap, and dance, where creativity is influenced by time constraints. Although the concept of improvisation, which includes both creativity and spontaneity (9), has been introduced, the sports context remains distinct from the arts.

The concept of “*creactability*” a construct that integrates creativity, quickness, and adaptability has been proposed as a more comprehensive framework for evaluating football performance than traditional measures of creativity. Athletes must be creative, spontaneous, and competitive within the formal constraints of the game, and “*creactability*” reflects these demands more effectively than isolated cognitive or physical indicators.

“*creactability*” comprises three core sub-factors quickness, creativity, and adaptability. It is further influenced by behavioral and psychological attributes such as analytical skills, positivity, composure, fundamental skills, and immersion. These factors collectively enhance a player's ability to respond effectively to rapidly changing play conditions an essential competency in modern football. In particular, quickness and adaptability facilitate rapid transitions between offensive and defensive roles, split-second decision-making, and flexible reactions to opponents' movements (10).

In traditional assessments, creativity in team sports has largely relied on subjective expert evaluations (6), while performance has often been measured using objective technical and physical indicators, such as number of shots, pass success rate, and ball possession (11–13), or total distance covered, maximum velocity, and high-intensity running (HIR) frequency (14–16). While these indicators remain valuable, advancements in training and conditioning have narrowed physical and technical differences between teams. As a result, breaking through defensive structures now increasingly requires unpredictable, intelligent play commonly referred to as spatial intelligence or creativity (8). Therefore, the need for more nuanced and multidimensional assessment tools like the “*creactability*” scale has become more pressing for talent identification and player development.

However, unlikely technical and physical indicators, using creativity as a measurement variable posed challenges due to difficulties in quantification, lack of uniform definition, etc (17). Few researchers have explored the perceptual and cognitive processes that underlie creative behavior in these performance contexts (7). For instance, eye movement recording was used to assess the visual search behaviors of skilled football players making decisions (18, 19), and verbal reports were employed to measure how players translate information from the visual system into appropriate creative cognitive processes and behaviors (20, 21).

**Figure 1:**
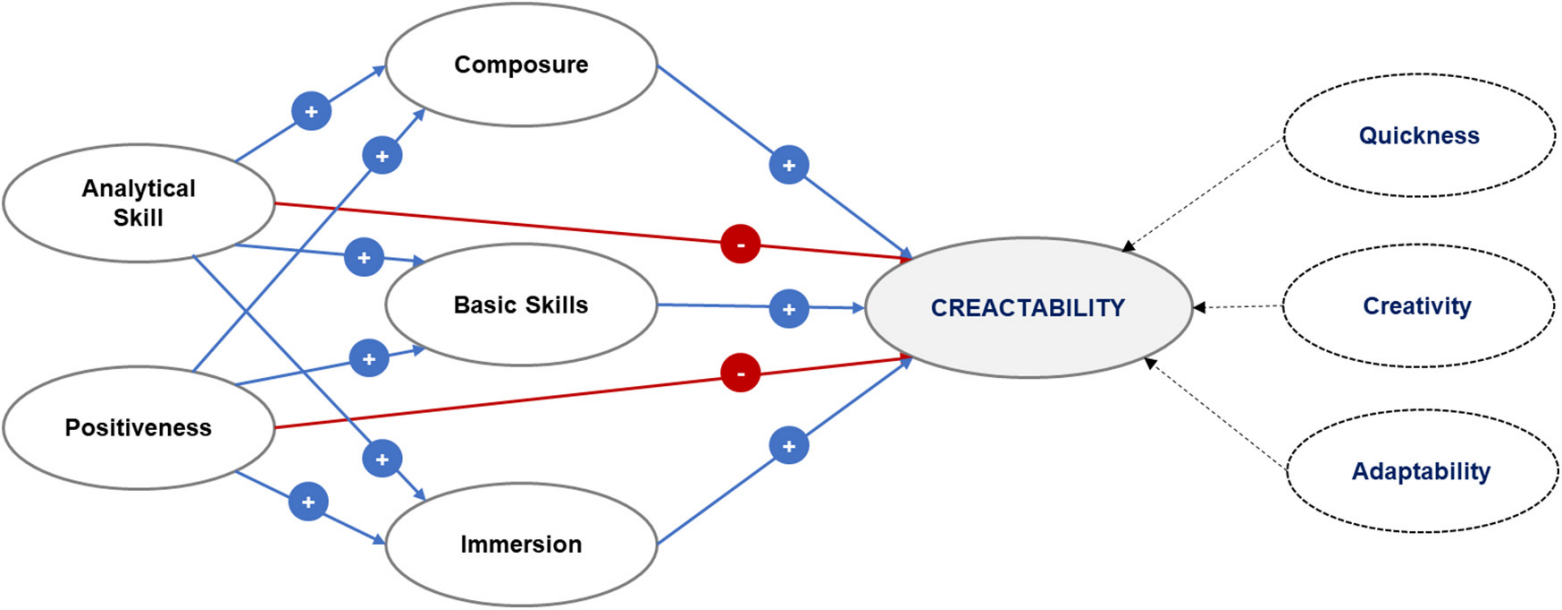
Research model for “creactability” (22).

In previous research, various perspectives on creativity in football have been explored. Lee, Kang & Kim (22) introduced the novel concept of “*creactability*” in sports using a grounded theory research method, where researchers develop a comprehensive theory based on the processes, behaviors, and interactions perceived by numerous research participants (23). Lee et al. (22) conducted qualitative analysis with Delphi survey techniques to establish a new model for creativity, followed by a quantitative analysis to examine hypotheses. They proposed that assessing creativity in football cannot be solely based on a specific indicator and introduced the novel concept of “*creactability*”. “*Creactability*” comprises three sub-factors (quickness, creativity, adaptability), and various factors (analytical skills, positivity, composure, basic skills, immersion) may positively or negatively influence “*creactability*”. The research model is depicted in Figure 1.

To assess the model's reliability, Lee et al. (22) employed various methods, including Cronbach's coefficient and Confirmatory Factor Analysis (CFA). However, there were limitations in generalizing the findings, and the methods for evaluating “*creactability*” have not been fully validated. An alternative approach could be the Rasch model, capable of converting data measured on a Likert scale into logit scores. The difficulty level is calculated using statistical methods, providing an objective and logical weight calculation instead of relying on subjective judgments. The Rasch model allows not only suitability analysis of the questions but also of dimensionality and the number of response categories, enabling a meaningful analysis beyond the scope of CFA. Previous research in physical education and sport psychology has demonstrated the utility of the Rasch model for instrument validation, particularly when assessing constructs that involve subjective judgment and multidimensional behaviors (24).

This study aimed to establish construct-related validity evidence for the “*creactability*” scale (22) in football by applying the Rasch model. Specific objectives included: (1) assessing whether each scale measures a unidimensional construct, (2) investigating Differential Item Functioning (DIF) based on team groups (i.e., the rankings of higher and lower teams), (3) ensuring a wide range of “*creactability*” item difficulty and personal ability (respondents “*creactability*” levels) for assessing changes, and 4) exploring differences in “*creactability*” across subscales (7-point scale).

## 2 Method

### 2.1 Participants

The dataset utilized in this study had been previously employed in research that applied Confirmatory Factor Analysis (CFA) methods to explore the construct-related validity of the “*creactability*” scale. To evaluate the utility of employing the “*creactability*” scale to assess the behavioral abilities of athletes in the sports field, football (soccer), the most popular sport over the world, were chosen as a sample. Using the developed scale, the survey was conducted from the professional football players, with the cooperation of the Korean Professional Football League (K-League). The coaches in K-League from 7 teams (Busan, Gangwon, Gwangju, Incheon, Jeonnam, Jeonbuk, Ulsan) assessed their players (*n* = 241), averaging 34.4 players per team, with goalkeepers excluded due to their distinct abilities and roles compared to other field players. Detailed participant information is provided in Table 1. All data used in this study were collected in 2011 (22).

**Table 1:** Information of the participants.

Characteristics	N	%
K-League team^a^		
A	40	16.6
B	33	13.7
C	33	13.7
D	29	12.0
E	38	15.8
F	35	14.5
G	33	13.7
Position^b^		
Forward	55	22.8
Midfielder	100	41.5
Defender	86	35.7
Total	241	100.0

This is the Table 1 legend.

^a^ K-League team = 7 teams of the Korean professional football league.

^b^ Positio*n* = positions of football players excluding goalkeepers.

### 2.2 Measures

The “*creactability*” scale was developed to measure factors related to the psychological behaviors of athletes in the sports field (22). The study used a total of 9 items, consisting of 3 items each for subfactors (i.e., quickness, creativity, and adaptability). A 7-point Likert scale was employed (i.e., not at all, not usually, not slightly, normal, slightly, usually, and very), and the contents of the items are presented in Table 2. Previous research validated the construct validity of the scale using CFA. According to accuracy indices like Chi-square, Goodness-of-Fit Index (GFI), and Comparative Fit Index (CFI), all criteria for a good fit were met. The internal consistency reliability ranged from .83 to .89 for Cronbach's alpha (*α*) and from .916 to .952 for factor loadings. Lee et al. (22) explored the relationship of the “*creactability*” scale with variables in sports, including analytical skill, positiveness, composure, basic skills, and immersion.

**Table 2:** Question items and scales for “creactability” (22).

Factor	Question Element
Quickness (3 questions)	He (she) makes a decision quickly
	He (she) reacts quickly
	He (she) figures out match situations quickly
Creativity (3 questions)	He (she) plays unexpected way from opponent
	He (she) tries new methods resolutely
	He (she) plays unique way compared to others
Adaptability (3 questions)	He (she) adapted new situation well
	He (she) endures stress well
	He (she) learns well

### 2.3 Data analysis using Rasch calibration

The “*creactability*” scale, designed to assess athletes in the sports field, is a 7-point Likert scale. Therefore, in this study, the Rasch rating scale model was applied. Rasch calibration was implemented in the following stages (25): (1) unidimensionality, (2) differential item functioning, (3) the easiness/difficulty levels of the items and the individual's “*creactability*” level, and (4) examining the levels in the subscales using the three-many-facet Rasch analysis.

#### 2.3.1 Unidimensionality

To validate the items of a scale, it's essential to measure a single construct. Deviations from the expected model may suggest that the items assess multiple domains within a multidimensional construct. The scale's unidimensionality can be evaluated by examining infit and outfit (mean squares) using Chi-square fit statistics. Infit and outfit values equaling 1 indicate a perfect fit with the model. However, if the fit statistics are below or above the specified criteria, this indicates potential issues with the over-fitting or under-fitting of the predicted model, respectively (26). Wright et al. (27) found 0.8–1.2 for high-stakes tests, 0.6–1.4 for rating-scale items, and 0.5–1.7 for clinical observations, indicating suitable fit. Alternatively, Linacre (28) suggests that values exceeding 2.0 indicate potential distortion or degradation, those between 1.5 and 2.0 reflect unproductive outcomes without degradation, values from 0.5–1.5 are considered productive, and values under 0.5 suggest reduced productivity and possibly exaggerated reliability estimates. In this study, the commonly used accuracy index criteria of 0.6–1.4 were applied (29, 30). When misfits in the items are identified, Rasch analysis should be re-conducted after removing the misfitting items. If the removal of an item affects the scale's accuracy, it can result in a reduction in the error rate of the model estimates after eliminating the misfitting item (31).

#### 2.3.2 Differential item functioning (DIF)

The examination of the influence of the team groups (i.e., the top 4 higher-ranked teams and 3 lower-ranked teams) on the endorsement of items in the “*creactability*” scale was conducted using Differential Item Functioning (DIF). This approach was chosen because any item might exhibit systematic bias towards a particular group, such as different team groups (32). In this study, uniform DIF was applied to investigate the variation between these two groups, representing the main effect. Non-uniform DIF, which examines interaction effects such as age and gender (33, 34), was not considered. This decision was made because the primary concern in developing new scales for this population was bias based on the rankings of higher and lower teams. Despite ongoing debates regarding the use of t-statistics criteria, it is a relatively common method employed to determine DIF. Therefore, the t-statistic with calculated logit scores was applied using WINSTEPS. The alpha (*α*) level was set at 0.01 after applying the Bonferroni correction technique due to multiple item comparisons (30, 35–37).

#### 2.3.3 Item difﬁculty and person ability

**Figure 2:**
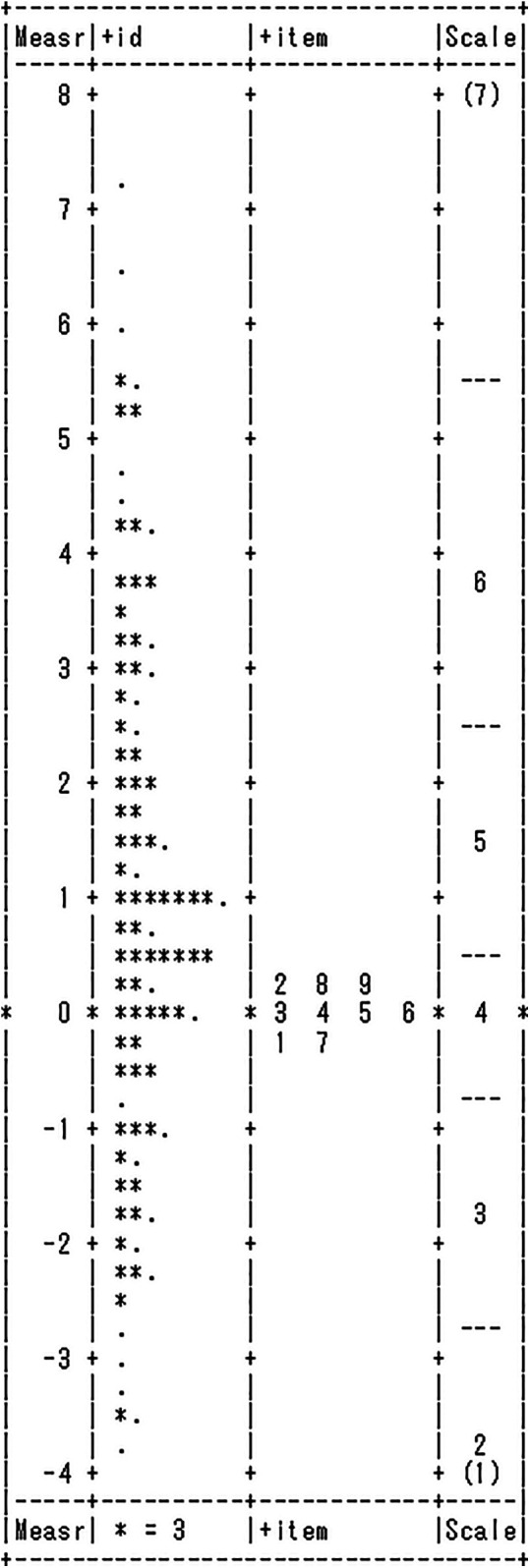
Item-person map of “*creactability*”.

With only well-fit items selected, the difficulty of “*creactability*” items and the person's level of “*creactability*” were calculated using the log-odds scale (logits), an interval scale. As described in the introduction, the logits of item difficulty and the person's level of “*creactability*” are independent and can be compared on a common scale (see Figure 2 for example). If the logit of the person's ability and the item difficulty are the same (e.g., logit = 0.30 for each), there is a 50% chance that the person will endorse the specific item.

#### 2.3.4 Many-facet Rasch model

The Rasch model offers an additional benefit in that it enables the analysis of subgroups or subscales' impacts by employing a multifaceted approach. This model allows for the computation of logit scores for distinct subcategories, thereby enabling their comparison using a uniform measurement unit (38, 39). The Rasch model has been successfully applied in sports and health-related fields (40–43). In this study, the “*creactability*” scale encompasses three subscales: quickness, creativity, and adaptability, and is structured on a 7-point scale. Therefore, three-facet Rasch analysis incorporating items, individuals, and the “*creactability*” subscales was utilized to meticulously examine the influence of “*creactability*” on the applied scores. For descriptive analysis, SPSS 26 (SPSS Inc., IL) was employed. The Rasch analyses were conducted using WINSTEPS Version 5.7.4 and FACETS Version 4.1.8, both of which are licensed, genuine software versions (44).

## 3 Results

### 3.1 Unidimensionality and model fit

In this study, a 7-point scale with 9 items was used to assess the unidimensionality of football players “*creactability*” (quickness, creativity, and adaptability). The infit and outfit statistics for each item are presented in Table 3. Although items 8 and 2 exhibited relatively high scores (indicating greater difficulty), the unidimensionality of all 9 items was confirmed as their infit and outfit values fell within the range of 0.7–1.3.

**Table 3:** Item difﬁculty and model ﬁt indices of “*creactability”.*

Item	Logits	Infit	Outfit
9	.28	.96	1.00
8	.23	1.19	1.24
2	.16	1.17	1.13
4	.12	.78	.74
6	−0.02	.90	.86
5	−0.05	.87	.86
3	−0.10	1.08	1.02
7	−0.25	.78	.80
1	−0.37	1.03	1.03

### 3.2 Differential item functioning (DIF)

To investigate the Differential Item Functioning (DIF) of scales based on team groups (i.e., the rankings of higher and lower teams), responses from 241 individuals were included in the study. None of the items in the position scales showed statistically significant DIF. In other words, no item was biased towards any specific team with *p* < 0.01 after Bonferroni correction.

### 3.3 Item difﬁculty and person ability

The distribution of respondent attributes and item difficulty levels for the “*creactability*” scale among football players presented in Figure 2. Both respondent attributes and item difficulty units are normalized to logits values and displayed on a single line. On this central logit line, the left side represents the attributes of 241 respondents, and the right side displays the difficulty of 9 items. Respondents and items positioned higher on this line indicate higher “*creactability*” for respondents and greater difficulty for the items. All 9 items were presented with a moderate level of difficulty. The person separation index (PSI) criterion value for the “*creactability*” scale of football players is 4 within a range of 0–7. Therefore, it indicates that the developed 9 items were effective in measuring the attributes of the 241 respondents.

### 3.4 Many-faceted Rasch results

**Figure 3:**
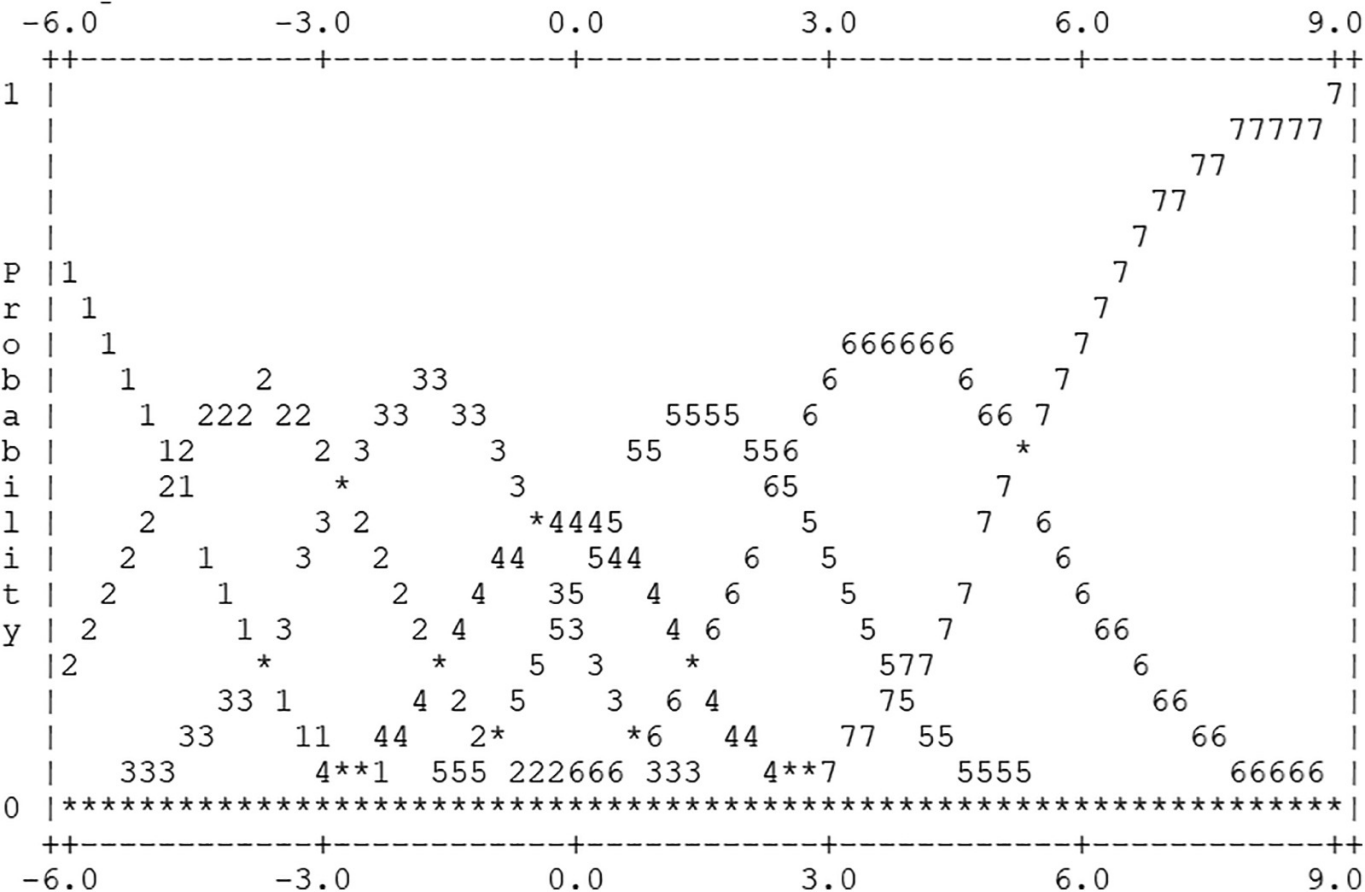
Probability curve of the item category.

The results of the three-facet Rasch analysis of “*creactability*” scale are summarized in Table 4. And the probability curve of item categories for the “*creactability*” 7-point scale in Figure 3. All subscales demonstrated good-ﬁt in both inﬁt and outﬁt between 0.69 and 1.28, respectively. The response rate for scales 4–5–6 showed 68%, and the outfit for respondents across all scales was found to be satisfactory, at 1.30 or lower. The step calibration, which is the numerical value of 6 points where the category probability curves of the 7 scales intersect, showed a progressively increasing trend in this study, confirming the appropriateness of the applied 7-point scale. However, further analysis is needed for optimal categorization.

**Table 4:** Logit scores and model ﬁt indices of “*creactability*”.

Scale	Frequency	Percent (%)	Avg. Measure	Outfit	Step-Calibration	Difference Step-Calibration
1	19	1	−3.18	1.00	None	
2	142	7	−2.18	1.28	−4.77	
3	398	18	−1.19	.90	−2.73	2.04
4	429	20	.14	.69	-0.51	2.22
5	610	28	1.34	.88	.33	0.84
6	441	20	3.04	1.02	2.42	2.09
7	130	6	4.91	1.10	5.26	2.84

## 4 Discussion

This study used the Rasch model to investigate the construct-related validity evidence of the “*creactability*” scale for athletes. The “*creactability*” in the field of sports must include elements of competition and victory in matches (9, 22). Therefore, it can be argued that the creativity within “*creactability*” is more suitably interpreted in a sports context rather than an artistic one. This study further examined the validity of the “*creactability*” scale developed by Lee et al. (22) using the advanced Rasch model.

After applying the Rasch rating model, the unidimensionality of all 9 items of the “*creactability*” scale was confirmed. Surprisingly, the DIF analysis revealed no biased items for specific groups within team groups (i.e., the rankings of higher and lower teams). This result could provide scientific evidence regarding the fair and accurate measurement of the conceptual construct of “*creactability*” in individual football players. All 9 items on the “*creactability*” scale were presented with a moderate level of difficulty. The person separation index (PSI) criterion value for the “*creactability*” scale of football players is 4 within a range of 1–7. Therefore, it indicates that the developed 9 items were effective in measuring the attributes of the 241 respondents using a 7-point scale category.

When applying the Likert scale, it is commonly known to use 5 or 7-point scales, and it has been reported that the reliability of the test does not increase further and shows a plateau when applying Likert scales larger than 7-point, thus it's not advisable to use scales larger than 7-point (45). Based on this, the process of developing scales based on classical test theory (CTT) can negatively affect the discriminative power and reliability of the scale due to the subjective determination of response categories by researchers (25). Consequently, many studies are applying a more specific and empirical validity verification method using item response theory (IRT), which takes into account discriminability and difficulty (25, 46, 47).

In IRT using the Rasch model, the determination of response categories is made using logit values between a person's attribute scores and item difficulty, and the probability curves of the likelihood of choosing a specific response category (48). Accordingly, this study utilized the Rasch model to analyze the appropriateness of response categories, and the results indicated that a 7-point category is suitable. The step calibration, which is the numerical value of the 6 points where the category probability curves of the 7-point scale intersect, showed a progressively increasing trend, confirming the appropriateness of the applied 7-point scale. However, as observed in Table 4 and Figure 3, the potential for categorization between 2 and 3 and 5–6 points can be identified. While new “*creactability*” measurement scales were developed, “optimal categorization” could also be adopted. This has been successfully applied in other studies utilizing the Rasch rating model (49–53). In the future, the function of categories (e.g., not at all, not usually, normal, usually, and very) should be investigated based on the Rasch model.

Previous studies have indicated that the creativity and adaptability of athletes positively impact their athletic performance (18, 54–56). Therefore, utilizing the “*creactability*” scale, which has been validated for football players, and coaches can provide an environment conducive to the development of players' decision-making ability. Coaches can lead more representative football-specific decision-making activities during training sessions (57). Also, it could serve as a crucial factor in assessing a player's football intelligence, which could be meaningfully utilized in scouting to recruit players. Even though the Rasch model has provided construct-related validity evidence for the “*creactability*” scale, more research using this developed scale is essential to explore its explanatory and predictive power among athletes from various sports. This necessity arises because the establishment of construct validity is an ongoing process (58).

## 5 Conclusions

This study examined the construct-related validity of the “*creactability*” scale for football players using the Rasch model. All items demonstrated unidimensionality, no DIF across team groups, and appropriate item difficulty and person separation. The 7-point response format was also validated. These results support the scale's validity for assessing creative and adaptive performance in football. Further validation and item refinement are recommended to broaden its applicability.

## Data Availability

The original contributions presented in the study are included in the article/Supplementary Material, further inquiries can be directed to the corresponding author/s.
